# Phenotype-Driven Variability in Longitudinal Body Composition Changes After a Very Low-Calorie Ketogenic Intervention: A Machine Learning Cluster Approach

**DOI:** 10.3390/jpm15060251

**Published:** 2025-06-14

**Authors:** Victor de la O, Begoña de Cuevillas, Miksa Henkrich, Barbara Vizmanos, Maitane Nuñez-Garcia, Ignacio Sajoux, Daniel de Luis, J. Alfredo Martínez

**Affiliations:** 1 Precision Nutrition and Cardiometabolic Health, IMDEA-Food Institute, Campus of International Excellence (CEI) UAM+CSIC, 28049 Madrid, Spain; victor.delao@alimentacion.imdea.org (V.d.l.O.); miksa.henkrich@gmail.com (M.H.); jalfredo.martinez@imdea.org (J.A.M.); 2 Faculty of Health Sciences, International University of La Rioja (UNIR), 26006 Logroño, Spain; 3 Translational Nutrition Sciences, Department of Human Reproduction, Growth and Child Development Clinics, Centro Universitario de Ciencias de la Salud (CUCS), Universidad de Guadalajara (UdeG), Guadalajara 44340, Mexico; bvizmanos@yahoo.com.mx; 4 Medical Department Pronokal Group, 08009 Barcelona, Spain; maitane.nunez@gmail.com (M.N.-G.); drsajoux@hotmail.com (I.S.); 5 Epigenomics in Endocrinology and Nutrition Group, Epigenomics Unit, Instituto de Investiga-ción Sanitaria de Santiago de Compostela (IDIS), Complejo Hospitalario Universitario de Santiago de Compostela (CHUS/SERGAS), 15706 Santiago de Compostela, Spain; 6 Center of Investigation of Endocrinology and Nutrition, Department of Endocrinology and Nutrition, Medicine School, Hospital Clínico Universitario, University of Valladolid, 47005 Valladolid, Spain; dluisro@saludcastillayleon.es; 7 Biomedical Research Centre for Obesity Physiopathology and Nutrition Network (CIBEROBN), Instituto de Salud Carlos III (ISCIII), 28029 Madrid, Spain

**Keywords:** body composition, very-low ketogenic diet, machine learning

## Abstract

**Background**: Obesity is a major global public health issue with no fully satisfactory solutions. Most nutritional interventions rely on caloric restriction, with varying degrees of success. Very low-calorie ketogenic diets (VLCKD) have demonstrated rapid and sustained weight loss by inducing ketone bodies through lipolysis, reducing appetite, and preserving lean mass while maintaining metabolic health. **Methods**: A prospective clinical study analyzed sociodemographic, anthropometric, and adherence data from 7775 patients undergoing a multidisciplinary nutritional single-arm intervention based on a commercial weight-loss program. This method, using protein preparations with a specific balanced nutritional profile, aimed to identify key predictors of weight-loss success and classify population phenotypes with shared baseline characteristics and weight-loss patterns to optimize treatment personalization. **Results**: Statistical and machine learning analyses revealed that male gender (−9.2 kg vs. −5.9 kg) and higher initial body weight (−8.9 kg vs. −4.0 kg) strongly predict greater weight loss on a VLCKD, while age has a lesser impact. Two distinct population clusters emerged, differing in age, sex, follow-up duration, and medical visits, demonstrating unique weight-loss success patterns. These clusters help define individualized strategies for optimizing outcomes. **Conclusions**: These findings translationally support associations with the efficacy of a multidisciplinary VLCK weight-loss program and highlight predictors of success. Recognizing variables such as sex, age, and initial weight enhances the potential for a precision-based approach in obesity management, enabling more tailored and effective treatments for diverse patient profiles and prescribe weight loss personalized recommendations.

## 1. Introduction

‘Globesity’ is a foremost challenge to societies and healthcare systems impacting the quality of life and well-being of millions of people [1]. Actually, obesity increases morbidity by rising the prevalence of chronic diseases such as type 2 diabetes [2], cardiovascular events [3], and certain types of cancer [4] among others. Moreover, strong and long-standing associations exist between excessive body weight and all-cause mortality [5].

In this context, obesity management requires a multidisciplinary approach to be effective [6]. The major component of a successful weight control program should be the consistent long-term maintenance of weight loss and body composition homeostasis as well as the prevention of undesirable body weight re-gain after body weight lowering [7]. Diverse high-intensity lifestyle intervention programs can reduce the initial weight of individuals with overweight and obesity by 5–10% [8]. Indeed, body weight loss in patients with obesity can be addressed by a variety of management strategies [9]. Thus, physical activity promotion and dietary emerging restriction are two fundamental therapeutical approaches, focusing on creating a negative energy balance and modulating body composition and excessive adiposity [10,11]. Also, some pharmacological treatments [8,12] or bariatric surgery [13] may provide additional support concerning body weight loss. Although caloric restriction is the major driver for weight loss, the distribution of macronutrients in a dietary intervention may benefit specific metabolic processes such as appetite, lipid turnover, thermogenesis, muscle preservation, inflammatory markers or hyperglycemia in adults with some impact on fat mass decreases [14,15].

Most of these body weight loss programs are based on inducing fat stores reduction and muscle mass preservation, recent evidence highlights that both caloric restriction and physical activity promotion are valid and effective interventions to achieve a durable negative energy balance, relevant for sustainable body weight loss [16,17], whose mechanisms and evolution need to be recognized and investigated in depth in order to characterize the metabolic processes involved, particularly those concerning the role of macronutrient distribution within energy-restricted diets.

In order to achieve healthy body weight loss, an individualized diet must be prescribed, within the wide variety of existing nutritional possibilities, that induce a state of negative energy balance that leads not only to weight loss but also to deal with obesity manifestations such as hyperglycemia, hypertension or hypercholesterolemia, which may need tailored prescriptions to produce personalized outcomes [18]. In the setting of structured diets, the very-low-calorie ketogenic diet (VLCKD) has shown promising results since it has been linked to reductions in body mass index (BMI), waist circumference, hemoglobin A1c (HbA1c), total cholesterol, triglycerides, as well as systolic and diastolic blood pressure [19,20], despite some putative side-effects have been claimed, which were never specifically proven [21].

The aim of the study was to characterize the factors influencing body weight loss and their contribution to the process, as well as to identify population subgroups of patients with obesity with different responses to a very low-calorie/moderate protein enriched ketogenic diet.

## 2. Material and Methods

### 2.1. Study Population

**Figure 1:**
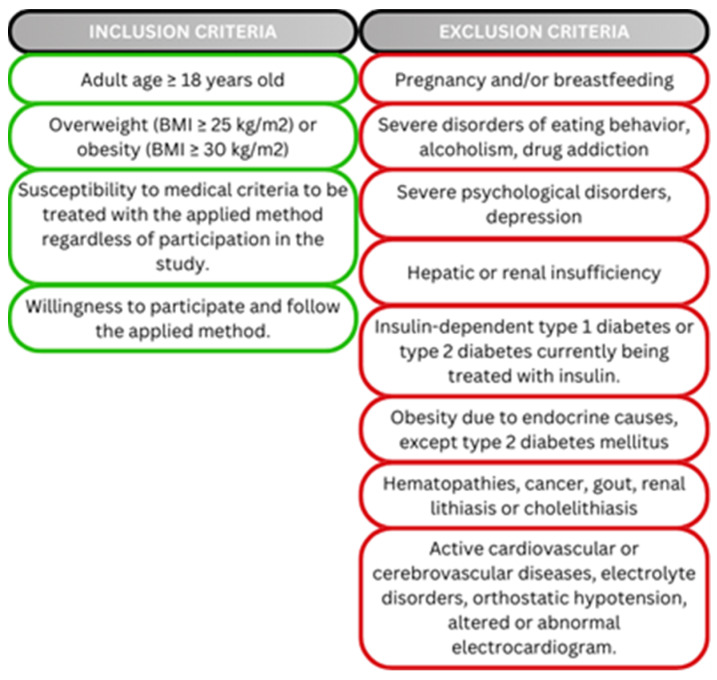
Inclusion and exclusion criteria.

A total of 7775 participants met all the inclusion and exclusion criteria (Figure 1) who were recruited between October 2020 and January 2022. Eligible participants were adult men and women aged more than 18 years old with overweight or obesity (BMI ≥ 25 kg/m^2^). Subjects of different nationalities were included: Spanish (6228 individuals), Portuguese (n = 381), Italian (n = 737), Belgian (n = 396) and Swiss (n = 33). This study was approved by the ethics committee of CEIm Pronokal Group, PNK-CONNECT-2020-01, 22 October 2020. Moreover, the research was performed in accordance with The Code of Ethics of the World Medical Association (Declaration of Helsinki) and related legislation applicable at the EU. All participants provided written informed consent before being included in the trial with specific inclusion and exclusion criteria (Figure 1).

### 2.2. Study Design

This study was a multicenter, prospective real-world intervention study based on a registry of 7775 patients undergoing treatment with a VLCKD under a standardized multidisciplinary method for body weight loss, which was implemented between March 2020–January 2022 following valid clinical criteria [22]. Of the 7775 participants initially enrolled, 362 individuals were excluded due to missing data on follow-up duration or adherence indicators. This resulted in a final analytical sample of 7413 participants, for whom complete demographic, anthropometric, and intervention-related data were available in order to adhere to a “par protocol” analytical approach. A literature search was performed on the main types of diets with changing macronutrient distribution for weight loss and body composition modulation, comparing their different effects on adiposity (Supplementary Table S1) to facilitate the interpretation of emerging results.

### 2.3. Multidisciplinary Nutritional Intervention

Patients followed a multidisciplinary obesity treatment program (the Pronokal or “PNK” method^®^) based on a VLCKD (energy-restricted moderately high protein diet), physical activity, dietary re-education and psycho-emotional support as described elsewhere [23] for different patients. No relevant clinical side effects were reported although ketonic breath and polyuria were often mentioned by participants. The intervention stage consists of a ketogenic diet, very low in calories (600–800 kcal/day), low in carbohydrates (<50 g daily from vegetables), and low in lipids (only 10 g of olive oil per day). The amount of high-biological-value proteins ranged between 0.8 and 1.2 g/kg of ideal body weight, to ensure meeting the minimal body requirements and to prevent the loss of lean mass. This method produces three ketogenic steps based on a high-biological-value protein preparation diet and natural foods. Each protein preparation contained 15 g protein, 4 g carbohydrates, and 3 g fat, and provided 90–100 kcal. In step 1, the patients eat high-biological-value protein preparations five times a day, and vegetables with low glycemic index. In step 2, one of the protein servings is substituted by a natural protein (e.g., meat and fish) either at lunch or at dinner. In step 3, a second serving of the natural protein substituted the second serving of biological protein preparation. Throughout these ketogenic steps, supplements of vitamins and minerals, such as K, Na, Mg, Ca, and omega-3 and omega-6 fatty acids, were provided in accordance with the international recommendations. In this method, the intervention stage is maintained until the patient loses most of the weight loss target (ideally 80%), although in this study the ketogenic step was maintained for 2 months and then the ‘key to success’ stage started with a no-ketogenic low-calorie diet. At this point, the patients underwent a progressive incorporation of different food groups to guarantee the long-term maintenance of the weight loss [24].

### 2.4. Foods Suitable for Ketosis

The core of the intervention program is ready-to-eat ketosis-friendly foods, low in carbohydrates and fat, with an adequate supply of protein from milk, eggs and soy [25]. The preparations have 80–110 kcal per serving, 1–6 g fat, 2–6 g carbohydrate and 15 g protein, formulated to enhance ketosis and prevent loss of lean mass. In addition, the preparations are supplemented with 50 mg of docosahexaenoic acid (DHA).

### 2.5. Nutritional Intervention Programming

**Figure 2:**
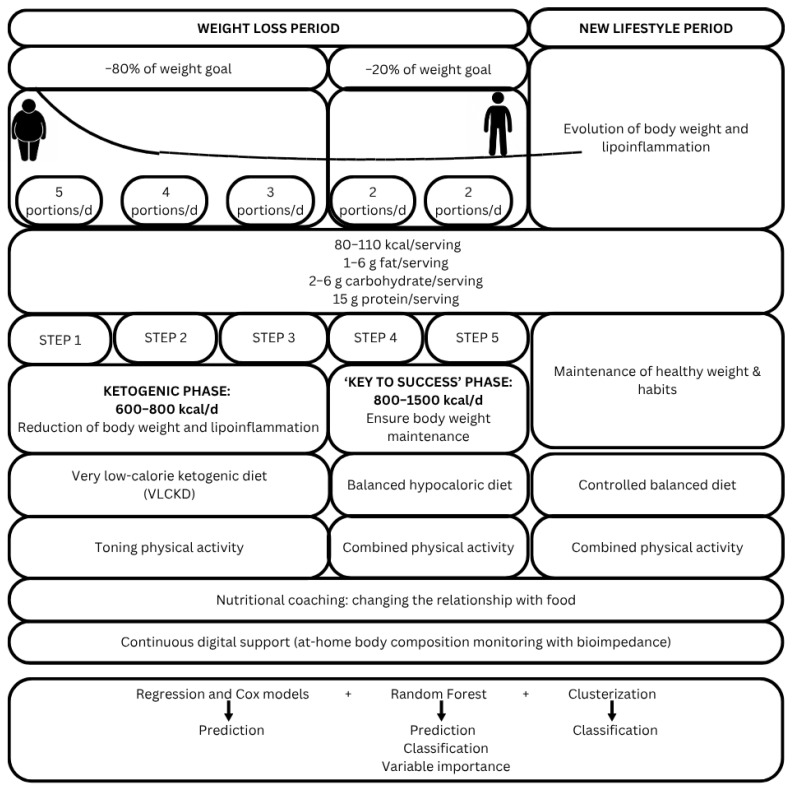
Programming and elements of the multidisciplinary intervention based on the PNK Approach.

The PNK-Method consists of two main phases (weight loss period and new lifestyle period). Period 1 includes five sequential steps, which conclude once the predefined goals are achieved. The components of the program and the multidisciplinary intervention involved in its implementation are detailed in Figure 2.

### 2.6. Duration of Intervention and Visits Schedule

Patients (a total of 7413 participants in the study were included) followed the nutritional program until the targeted weight was achieved. During the intervention, medical visits were scheduled every 15, 21 or 30 days to prescribed a treatment and monitoring to ensure diet adherence and to monitor possible side effects, which were examined by specialized medical staff. Typically, the analyzed group resulted in 14–17 weeks of intervention. During the visits, patients received instructions on the nutritional guidelines, individualized counseling and support, and were encouraged and advised to practice the prescribed physical activity pattern.

### 2.7. Lifestyle Assessments and Anthropometric Measurements

Sociodemographic, clinical, diet adherence and lifestyle data were collected through digital platforms devised for health professionals involved in the prescription and administration of the intervention (physicians and nutritionists), as well as an application for patients which was validated by an external monitorization. The program collected data on body weight (kg), BMI (kg/m^2^), total body fat (%), visceral fat (%), muscle mass (%) that were obtained by using a LifeVit BL-2500 bioimpedance scale (LifeVit, Barcelona, Spain), at baseline, 1 time/week and until the end of the program.

### 2.8. Statistical Analysis

A descriptive analysis concerning anthropometric data across sex-, age-, and BMI- specific groups was performed. The normality of the variables was screened using the Shapiro–Wilk test. Descriptive statistics were given as mean and standard deviation (SD), and differences were assessed by Student’s t test for continuous variables. The χ^2^ test for categorical variables and Fisher’s exact test for categorical variables with a prevalence of less than 5% were implemented.

Regression models for predicting outcomes, Cox regression for time roles, and Random Forest for assessing the graded importance of relevant variables and hierarchical clustering to categorize subjects’ outcomes were implemented. Multiple linear regression models were fitted to determine the combined influence of several variables on different metrics of weight loss success. The variables used in the regression models were age (years), sex (male/female) cumulative expenses × 1000 (€), number of visits (continuous), and diet restart (yes/no). Model 1 studied the association of these variables with body weight (kg), model 2 with BMI (kg/m^2^), model 3 with total body fat (%), model 4 with muscle mass (%). Also, logistic models were fitted using recognized cut-off for body compartment (%) analyses.

Cox proportional hazards models were estimated separately for each outcome of interest, with survival time defined from study entry to event occurrence. The time origin was set at baseline, and follow-up was measured in weeks from study entry to either event or censoring.

Unsupervised classification machine learning analyses, devised for clustering performed using Ward’s hierarchical clustering method (STATA’s cluster linkage function), were performed to identify population subgroups revealing possible phenotypical differences in weight loss and to detect traits associated with these differences.

The conducted hierarchical cluster analysis was able to identify distinct subgroups of participants based on demographic, behavioral, anthropometric, and clinical variables, as well as treatment adherence indicators.

The clustering model encompassed sociodemographic and intervention-related variables, including sex, age, intervention group, total days in the program, temporal marker of follow-up, number of doses consumed, number of doses missed, daily dose rate, and accumulated treatment cost in addition to intervention outcomes. Thus, baseline anthropometric and body composition variables included initial weight, initial body mass index, initial body water, initial muscle mass, initial basal metabolic rate, initial visceral fat, and initial body fat percentage. In addition, a set of change-related variables reflecting the evolution throughout the intervention were included: absolute changes in weight, BMI, muscle mass, BMR, visceral fat, and body fat percentage. Corresponding percentage changes were also used: percentage change in weight, BMI, muscle mass, BMR, visceral fat, and body fat percentage. Finally, binary indicators of clinical success, defined as a reduction of ≥10% from baseline, were incorporated: success in weight loss ≥ 10% and success in body fat loss ≥ 10%. The selection of a 2 clusters solution was determined by elbow and silhouette analyses.

Subsequently, a Random Forest model was used for categorization and prediction purposes related to the clusters established in the previous step and for quantification of the contributing role to the model using *rforest* in STATA version 12.1 (StataCorp, College Station, TX, USA) as well as to assess the contributions of relevant variables [26]. The contribution of each variable to the clustering model was characterized and revealed a maximum contribution (value of 1 on a scale from 0 to 1, from least to greatest contribution) to the formation of the clusters.

All the presented *p*-values are two-tailed and were considered statistically significant at *p* < 0.05. Data was analyzed using STATA version 16 (StataCorp LLC, College Station, TX, USA).

## 3. Results

### 3.1. Characteristics of the Study Population

This weight loss response characterization study employed a multidisciplinary intervention of a VLCKD followed by a balanced hypocaloric diet, physical exercise and psychoemotional support. The anthropometric characteristics of the participants, separately analyzed by sex, age, and BMI are reported (Table 1). The average intervention period is about 14–17 weeks to achieve 80% of the targeted weight loss.

Age showed no statistically significant differences when classifying the population by sex (*p* = 0.297), however, in both treatment follow-up and anthropometric variables, highly significant differences were observed (*p* < 0.001), with better results observed in men (Table 1). Women showed lower initial weight, but higher body fat and lower visceral and muscle mass than men. When classifying the population by median age, it was observed that the older group had slightly lower baseline weight and muscle percentage values (*p* < 0.001), as well as a slight relative increase in baseline lean mass (*p* = 0.022) and a 3-week longer follow-up (*p* < 0.001). Older participants had lower baseline body weight, but higher body fat, visceral fat and lower muscle mass at baseline. Patients with a higher BMI (≥30 kg/m^2^) did not show differences in age but did show differences in the rest of the variables, presenting worse baseline anthropometric values, but better response to treatment (*p* < 0.001). As expected, the group with higher BMI observed higher basal.

### 3.2. Prediction Models: Multiple Regression Models

Several multiple regression models fitted with appropriated covariables and confounding factors. Model 1 explained 35% of the observed body weight loss variance, baseline body weight and number of medical visits predict greater body weight loss success with highly significant *p*-values, while cumulative expenditure and especially diet restart were correlated with lower body weight loss (Table 2). High initial BMI and number of medical visits showed the greatest contributions to BMI reduction success (Model 2), while female sex, and diet restart predicted lower success. The model explained 36% of the observed variance in BMI reduction. Also, high initial fat mass and number of visits showed a marked positive association with fat loss, while female sex, restart and, although slightly, age showed negative associations with it, in a model that explains 35% of the observed variance (Model 3).

Regarding changes in the proportion of body muscle, model 4 showed that greater baseline muscle mass, age, female sex, and diet restart correlate with a decrease in muscle percentage, while greater adherence predicted a greater relative increase in muscle in a model that explains 35% of the observed variance (Table 2).

Several logistic regression analyses using recognized threshold of success were fitted. Model 1, which explains 24% of the variance observed in the loss of at least 10 kg of body weight, baseline body weight and number of visits predicted greater success in losing at least 10 kg of body weight with highly significant (*p* < 0.001), while the influence of sex and age was not statistically significant. According to Model 2, which explains 24% of the variance observed in the loss of at least 3 kg/m^2^ BMI, baseline BMI, number of visits and to a lesser degree, cumulative expenditure showed a significant positive influence on success (*p* < 0.001), while female sex is correlated with lower success (*p* = 0.001).

Model 3, that explains 11% of the variance observed in the loss of at least 5% of fat mass weight, baseline fat mass (*p* < 0.001), number of visits (*p* < 0.001) and cumulative expenditure (*p* = 0.004) showed a highly significant positive influence on success, while older age (*p* = 0.006) and women (*p* < 0.001) correlate with lower success. Based on the Model 4, that explains 18% of the variance observed in the increased of at least 2% of the body muscle, cumulative expenditure and number of visits exert a positive influence on success (*p* < 0.001), while baseline muscle mass, older age, female sex, and restart predict a lower relative increase in body muscle (Table 3).

The hazard ratio (HR) analysis based on Cox regression models to assess the role of time and adherence to the treatment revealed significant differences between clusters 1 and 2 across key outcomes. For the event of ≥10 kg weight reduction after restart, individuals in cluster 2 exhibited a markedly higher risk (HR = 2.26; 95% CI = 1.73–2.95; *p* < 0.001) compared to cluster 1. Similarly, for achieving ≥ 5% weight loss, cluster 2 showed an increased hazard (HR = 1.27; 95% CI = 1.09–1.47; *p* = 0.002). In contrast, cluster 1 demonstrated stronger associations with baseline variables such as fat mass and BMI, with higher HRs observed for these predictors. Regarding ≥ 3-unit BMI reduction, cluster 2 had a significantly elevated risk (HR = 1.69; 95% CI = 1.35–2.12; *p* < 0.001), indicating greater responsiveness or risk in this subgroup. Notably, for muscle mass increase ≥ 2 kg, cluster 2 again showed increased hazard (HR = 1.39; 95% CI = 1.17–1.65; *p* < 0.001), while cluster 1 had stronger protective effects linked to baseline muscle mass. The interaction terms between cluster membership and days in treatment were statistically significant (*p*-interaction < 0.001), suggesting that treatment duration influences outcomes differently across clusters. These findings highlight distinct risk profiles and treatment responses, with cluster 2 consistently showing higher hazards for weight and BMI reduction-related outcomes, whereas cluster 1 appears more influenced by baseline metabolic characteristics (Supplemental Table S2).

Following unsupervised machine learning analyses two population subgroups or clusters were identified (Table 4), where the differences between the two groups are highly significant. Cluster 1 is larger in size, and is composed mostly of females, with an older average age (n = 5528, 74.6% of the population, females = 93.1%, mean age = 47.5 years), while cluster 2 is mostly male with a slightly younger average age (n = 1885, 25.4% of the population, females = 35.2%, mean age = 44.8 years).

Both clusters showed similar duration of follow-up, however, individuals in cluster 2 consume slightly more doses per day on average. Members of the second cluster started the intervention with a higher body weight and BMI (98.7 kg, 32.5 kg/m^2^ vs. 78 kg, 29.4 kg/m^2^). At the same time, these patients lose markedly more body weight (9.9 kg vs. 5 kg weight loss), total fat mass (14.9% vs. 8.2% reduction in total fat) and visceral fat mass (12.7% vs. 6.5% reduction in visceral fat), while showing a greater increase in muscle percentage (13.3% vs. 6.9% increase in muscle percentage) (Table 4).

The contribution of each variable to the clustering model was characterized using a Random Forest algorithm, which revealed a maximum contribution (value of 1 on a scale from 0 to 1, from least to greatest contribution) of age to the formation of the clusters (Table 4). After age, modifiable adherence factors such as total follow-up days, number of visits, or accumulated expenses hold greater importance, followed by anthropometric factors such as initial weight and BMI, sex, and weight loss success metrics.

### 3.3. Body Weight and Body Composition Changes by Cluster

**Figure 3:**
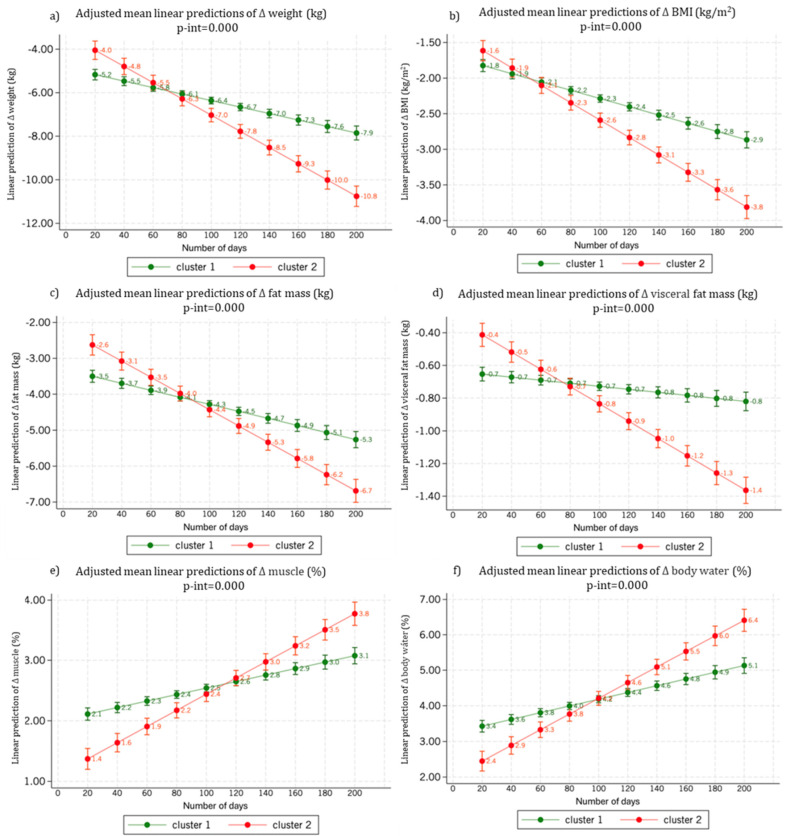
Conventional predictions of weight loss (**a**), BMI (**b**), fat mass (**c**) and visceral fat mass (**d**), as well as muscle (**e**) and body water gain (**f**), using a linear regression model adjusted for cluster, number of visits, initial weight, sex, accumulated expenses, restart, and the baseline value of the dependent variable.

In the predictions of body weight loss and reduction in BMI, individuals in cluster 2, starting from higher values, are expected to reach lower weight and BMI than individuals classified in cluster 1 within 80 days, and in the long term, they will lose significantly more weight, reducing their BMI to a greater extent (Figure 3a,b).

Regarding body composition, individuals in cluster 2, entering the intervention with more total fat and visceral fat (Figure 3c,d), will lose more adipose tissue in both compartments. Additionally, the significantly steeper slope of the line regression corresponding to visceral fat loss in cluster 2 compared to cluster 1 is noteworthy: those in cluster 2 will achieve a significantly greater loss of visceral fat throughout the intervention.

Concerning changes in muscle percentage relative to total body weight and water weight (Figure 3e,f), individuals in cluster 2 start with a generally lower muscle proportion and body water compared to cluster 1, but they increase their muscle mass percentage and water composition throughout the intervention, mainly due to the greater fat mass loss in this group.

## 4. Discussion

Obesity constitutes a public health burden and an outstanding clinical challenge with a worldwide high impact, whose etiology and treatment are highly complex, being dependent on many factors [27]. Indeed, previous prediction of weight loss outcomes may result in better patient management in a personalized and precision manner [28]. Therefore, featuring individualized characterization is permitting to focus personalized treatments by sex, age, and anticipate adherence to provide precision tailored obesity management. In this context, data-driven obesotyping, such as the identification of predictors of body weight loss and baseline traits of obese individuals or population subgroups is valuable information, as well as the forecast of weight loss allows adjustments in the personalization of treatments concerning excess weight in order to implement personalized medicine. A modest body weight loss (5−10%) is considered to be clinically significant as it reduces cardiometabolic risk factors and reduces obesity-related comorbidities in subjects with excessive adiposity [29], which is achieved differently depending on sex, age and initial adiposity (BMI). Nevertheless, significant body weight loss (≥ 10%) may have further beneficial effects [30,31], whose prediction depends on baseline phenotypical features may be useful for individual management and personalized prescriptions.

Indeed, obesity, accompanied by excessive adiposity and low degree inflammation, is a chronic syndrome that is important for health and quality of life [32]. Excess body weight is related to various complications and morbidities, as well as premature mortality [5]. Moreover, obesity is associated with a wide range of complications and comorbidities that impact both physical and mental health [33]. Common adverse conditions accompanying excessive body weight include cardiovascular manifestations and diseases [34], hyperglycemia [35], hypertension [36], sleep apnea [37], and certain types of cancer [38]. Additionally, obesity increases the risk of developing joint diseases such as osteoarthritis, as well as related to psychological disorders like depression and anxiety [39]. These conditions contribute to higher mortality rates and reduced quality of life in affected individuals [40]. The complex interaction among these co-medical complications and comorbidities makes obesity a multifactorial issue that is challenging to manage clinically, which require individual phenotypical and manifestations, where macronutrient distribution may have a role to impact specifically some obesity-associated and metabolic traits/disturbances [41].

The therapeutic strategies available for the treatment of obesity usually follow a stepwise order, inducing weight loss through energy restriction and programmed increased physical activity as the first treatment guidelines, and pharmacological or surgical treatment for refractory cases [42]. Different types of diets designed with differences in macronutrient content (low-calorie diet [LCD], very low-calorie diet [VLCD], VLCKD), selection of certain food groups (Mediterranean Diet) and time manipulation (intermittent diet) are currently considered to manage overweight and obesity [18] as discussed in the Supplemental Table S1, moreover, the magnitude of weight loss observed in our cohort is comparable to or greater than that reported for other dietary strategies of similar duration, highlighting the potency of the VLCKD approach. Some previous studies have suggested that VLCKD commercial programs such as PNK-method may be effective tools to manage overweight and obesity [43]. VLCKD diets are able to induce weight loss through different mechanisms involving appetite control, body composition redistribution, lipid breakdown and polyuria, which are affected by various factors such as age, gender, physical activity, BMI, genetics, etc. [44], whose reported side-effect were of low clinical relevance. In any case, both in terms of efficiency and efficacity this program achieves better outcomes than comparable trials concerning fat mass reduction and muscle preservation [22]. Indeed, ketogenic diets low in carbohydrates and moderately high in proteins have been shown as suitable for inducing healthy body composition changes with achievable goals [45,46,47,48,49,50,51,52,53,54,55,56,57].

There are numerous factors that can influence body weight [58]. Diverse studies show that males reported losing more weight than females when following the same diet [59]. Furthermore, the age of the observed individuals affected the rate and effectiveness of weight reduction of age [60]. Moreover, a fundamental factor in body weight loss is the initial body weight or BMI [61]. Several studies have shown that people with a higher BMI are more likely to have a greater probability of body weight loss [62,63]. On the other hand, body fat distribution is clinically important. Thus, body weight loss via diet and/or physical exercise is able to improve the regulation of free fatty acid metabolism, where the distribution of body fat mass is implicated [64,65]. The baseline fat mass (% of body weight) and baseline visceral fat (% of body weight) were comparable when using 47 years old cut-off but followed expected treats associated to aging. While baseline muscle mass (% of body weight) was lower in older subjects which could be partially explained by the differences in baseline weight. The fact that we compared the percentage of body weight may hinder the analysis of the data but this clear that the treatment reduces more body fat that lean mass in absolute values as a consequence of the treatment and apparently the percentual contribution of lean mass was preserved and proportionally increased.

The person’s pathophysiological condition and regional body fat distribution can also significantly affect weight loss [5]. Hormonal disturbances, insulin resistance, chronic inflammation and other metabolic disorders are key factors that can complicate weight loss [66], which may require specific dietary macronutrient distribution in the dietary prescription to achieve specific benefits.

In recent years, the role of the intestinal microbiota in the development and treatment of obesity has received increasing attention. Several studies suggest that individual variability in gut microbiome composition may influence both obesity risk and responses to weight-loss interventions, including dietary strategies such as VLCKD [67]. In this context, although gut microbiota was not assessed in our study, it is a potentially relevant factor for understanding personalized responses to dietary interventions. Similarly, genetic factors also play a key role in weight loss, influencing metabolism, dietary responsiveness, and fat storage predisposition [66,68]. While these personalized factors are important, behavioral components such as diet, physical activity, and lifestyle remain critical determinants of successful weight management [69]. Bariatric surgery, although not addressed in this study, represents a more invasive alternative for weight loss that also involves physiological mechanisms such as gut microbiota alterations [70].

In this context, the PNK method is a slimming diet based on a ketogenic diet, low in carbohydrates and fats, with an adequate protein intake that allows losing fat and preserving muscle mass [71]. The rapid and sustained body weight and fat mass loss induced by the VLCKD is often associated with improvements in the psychological well-being parameters [24].

Randomized controlled trials based on low calorie/low carbohydrate ketogenic diets have demonstrated weight reduction over 6 months and improvement of other metabolism-related disorders such as dyslipidemia, hypertension or insulin resistance [72]. Several reviews and reports have confirmed that there is insufficient information to conclude that these types of diets are not safe, but that they are effective for body weight loss [21,73,74]. VLCKD has been proposed as a valid dietary strategy to reduce body fat while preserving muscle mass [75,76]. These data have been confirmed with current cohort of patients since VLCKD diets may produce significant body weight losses by attenuating the increase in ghrelin secretion and the sensation of hunger [77], but also by addressing homeorhetic flexibility.

Although we acknowledge the inherent limitations of BIA in estimating visceral fat and skeletal muscle mass, previous studies have supported value and utility in clinical and epidemiological settings. Additionally, the inclusion of absolute body composition values in our tables and figures allows for a better interpretation of longitudinal changes, facilitating inter-individual comparisons and future estimations by other researchers [78,79].

Our weight loss clustering models estimate the final body weight after about three- four months based on initial body composition variables such as fat mass and fat-free mass. In contrast, weight dynamics have been modeled using an energy balance approach based on caloric intake and expenditure [80], and several clinically validated thermodynamic models incorporating digital tools have also been developed and re-evaluated [81]. While all described models aim to predict weight loss, ours stands out for valid simplicity and direct focus on body composition, making it practical for clinical settings without continuous monitoring of energy intake and expenditure being based on cluster analysis rather than algorithms. The results of the different clusters may have implications for tailoring interventions in clinical settings. Such differentiation may contribute to optimizing outcomes and resource allocation in clinical practice involving sex, age, baseline phenotype characteristics with an impact on precision recommendations. Indeed, the unsupervised clustering is exploratory in nature but provided valuable insights to the analyses, which need to be examined cautiously as well as the issue that proper control group is lacking and that participants baseline data were used as a reference for analyzing observational outcomes under a ketogenic dietary regimen. Despite the sample size, type I and II errors can not be disregarded either inference and causal alternative interpretations, but the fact that baseline data are incorporated to the model provide some individual’s variability in biological markers concerning weight loss assessment.

Several limitations may have concurred in the current analysis. First, the multicenter nature of the study, as well as a certain level of customization of the diets administered in terms of the exact products consumed and the proteins and vegetables accompanying them may have introduced biases in the data that we had not recorded. Second, we also did not collect data on prescribed physical exercise regimes which were advised with a common standard counseling and details of psychoemotional counseling sessions, which implies the possible loss of influential factors in the results, and this paper does not place greater emphasis on the analysis and interpretation of patient adherence data. Third, this was a single-arm study in a real-world clinical setting without a control group. This design makes it difficult to directly compare our results to other weight-loss methods. To partially address this, we compiled a reference table as a suitable comparison concerning body composition modifications induced by slimming diets. Fourth, several data were not recorded or were collected only from a limited number of patients, such as hip circumference, waist-to-hip ratio, comorbidities, or medication consumption, despite potential contribution to the model. Fifth, the lack of long-term follow-up data after the VLCKD intervention. Although a maintenance phase was initiated, outcomes from this period were not analyzed, and future research should track participants over time to evaluate weight regain and the sustainability of changes in body composition and metabolic health. Another limitation is the attrition and variability in follow-up duration across participants. Individuals with incomplete follow-up or adherence data were excluded, which may introduce selection bias and limit the generalizability of the findings, which was encompassed by using Cox-regression analyses (Supplementary Table S2) in order to account for time and adherence issues. Finally, data are often extrapolated concerning body weight loss to improvements in the mobility disorders associated with obesity, such as diabetes mellitus, hypertension, hyperlipidemia, etc. However, they also have strengths, such as the large sample size of the population studied, the statistical analysis methodology as well as the standardized intervention methodology with this VLCKD diet as well as that compared with 10 different weight lowering dietary interventions with a different macronutrient distribution with comparable duration to ascertain the value of ketogenic intervention.

An investigation concerning weight loss interventions evidenced uncommon changes involving gut microbiota profiles [82]. Furthermore, metabolic bariatric surgery showed favorable postoperative biochemical outcomes [83]. Although beyond the scope of our study, such approaches highlight the range of therapeutic strategies available—from nutritional to surgical—and the importance of tailoring interventions to individual patient characteristics and clinical scenarios highlighting that obesity management is benefiting by personalized precision approaches accounting integrated variables.

## 5. Conclusions

The described multidisciplinary method based on a very low-calorie ketogenic diet followed by a balanced hypocaloric diet, relying on specifically formulated ketogenic- profile foods and including physical exercise and psycho-emotional support, demonstrate an association with marked and rapid weight loss with healthy improvements in body composition, however, causality should be cautiously toned down as this is a longitudinal single-arm intervention study. Specifically, the trial resulted in a pronounced reduction in total and visceral fat with muscle mass maintenance/preservation. The success of the treatment is influenced by several factors, especially sex, age and baseline body weight, as confirmed by descriptive data and machine learning cluster analyses.

This study employs innovative factorial analyses and cluster modeling with robust unsupervised machine learning approach, providing valuable insights into personalized weight, fat, and muscle loss predictions. This data-driven obesotyping approach enhances previous models by incorporating body composition variables, offering a more precise classification for obesity management.

## Figures and Tables

**Table 1:** General characteristics and body composition changes following VLCKD in the population categorized by sex, age and BMI.

	Sex			Age			Baseline BMI		
	Men	Women	*p*-Value	<47 y.o.	≥47 y.o.	*p*-Value	<30 kg/m^2^	≥30 kg/m^2^	*p*-Value
**n**	**1536**	**5700**		**3604**	**3632**		**3768**	**3468**	
**Age (years)**	47.2 (10.6)	46.9 (10.4)	0.297	38.7 (6.7)	55.1 (6.3)	**<0.001**	46.8 (10.4)	47.1 (10.5)	0.297
**Women (%)**	-	-	-	79.4	78.2	0.205	85.7	72.4	**<0.001**
**Total days of follow-up (days)**	99.6 (95.9)	115.4 (112.3)	**<0.001**	100.4 (98.7)	123.6 (117.6)	**<0.001**	95.2 (101.2)	127.6 (113.9)	**<0.001**
**Number of visits (n)**	4.9 (4.0)	5.7 (4.6)	**<0.001**	5.1 (4.1)	6.0 (4.9)	**<0.001**	4.8 (4.0)	6.2 (4.9)	**<0.001**
**Doses per day (n)**	4.95 (0.8)	4.82 (0.7)	**<0.001**	4.9 (0.7)	4.8 (0.8)	**<0.001**	4.8 (0.8)	4.9 (0.7)	**<0.001**
**Total doses (n)**	86.4 (27.6)	83.3 (24.3)	**<0.001**	84.6 (25.3)	83.4 (24.9)	**0.036**	82.2 (23.0)	85.6 (26.7)	**<0.001**
**Accumulated expense (€)**	1859.2 (1489.5)	2047.6 (1710.6)	**<0.001**	1840.4 (1486.5)	2173.6 (1815.2)	**<0.001**	1669.7 (1387.5)	2318.7 (1835.4)	**0.002**
**Baseline weight (kg)**	98.2 (14.6)	80.0 (9.8)	**<0.001**	84.5 (13.6)	83.1 (12.9)	**<0.001**	80.0 (9.8)	98.2 (14.6)	**<0.001**
**Δ weight (kg)**	−9.2 (7.4)	−5.9 (5.5)	**<0.001**	−6.7 (6.3)	−6.5 (6.0)	0.372	−4.0 (3.6)	−8.9 (7.0)	**<0.001**
**Baseline BMI (kg/m^2^)**	32.3 (4.4)	30.0 (3.2)	**<0.001**	30.4 (3.8)	30.5 (3.5)	0.438	27.6 (1.4)	33.1 (3.0)	**<0.001**
**Δ BMI (kg/m^2^)**	−3.0 (2.5)	−2.2 (2.2)	**<0.001**	−2.4 (2.4)	−2.4 (2.2)	0.786	−1.5 (1.3)	−3.3 (2.7)	**<0.001**
**Baseline fat mass (% of BW)**	36.7 (7.4)	41.5 (5.2)	**<0.001**	40.3 (6.1)	40.6 (6.1)	**0.022**	36.4 (4.5)	44.4 (4.7)	**<0.001**
**Δ fat mass (% of BW)**	−5.9 (5.0)	−3.9 (3.9)	**<0.001**	−4.4 (4.2)	−4.3 (4.2)	0.389	−2.7 (2.8)	−5.9 (4.8)	**<0.001**
**Baseline visceral fat (% of BW)**	12.4 (2.8)	5.9 (1.3)	**<0.001**	7.3 (3.2)	7.4 (3.2)	0.695	6.1 (1.9)	8.5 (3.7)	**<0.001**
**Δ visceral fat (% of BW)**	−1.7 (1.6)	−0.5 (1.0)	**<0.001**	−0.8 (1.3)	−0.8 (1.2)	0.553	−0.4 (0.6)	−1.1 (1.5)	**<0.001**
**Baseline muscle mass (% of BW)**	32.0 (5.1)	29.4 (3.6)	**<0.001**	31.3 (3.9)	28.6 (3.8)	**<0.001**	32.3 (3.4)	27.7 (3.4)	**<0.001**
**Δ muscle (% of BW)**	3.7 (3.1)	2.2 (2.3)	**<0.001**	2.5 (2.6)	2.5 (2.5)	0.365	1.6 (1.7)	3.4 (2.9)	**<0.001**

Variables are shown as mean (SD) or as proportion (%). Continuous variables were compared using *t*-test. Categorical variables were compared using Chi-squared test or exact Fisher test. Data are shown separately by sex (men vs. women), by age (below vs. above median age 47), and by BMI category (<30 vs. ≥30), with *p*-values for each comparison. Significant values are in bold font. BMI, Body Mass Index; BW, Body Weight.

**Table 2:** Linear regression models of body weight, BMI, fat mass loss and muscle gain concerning different anthropometric and body composition outcomes.

	β	C.I.	*p*-Value	R^2^
**Model 1**	**Δ Body weight (kg)**			0.35
Baseline body weight (kg)	−0.21	−0.22; −0.19	**<0.001**	
Age (years)	0.01	−0.01; 0.02	0.268	
Women	−0.16	−0.51; 0 0.2	0.383	
Accumulated expense (1000€)	−0.46	−0.58; −0.33	**<0.001**	
Number of visits (n)	−0.28	−0.33; −0.23	**<0.001**	
**Model 2**	**Δ BMI (kg/m^2^)**			0.36
Baseline BMI (kg/m^2^)	−0.29	−0.30; −0.28	**<0.001**	
Age (years)	0.01	0.00; 0.01	**<0.001**	
Women	0.24	0.14; 0.35	**<0.001**	
Accumulated expense (1000€)	−0.10	−0.18; −0.09	**<0.001**	
Number of visits (n)	−0.10	−0.12; −0.08	**<0.001**	
**Model 3**	**Δ Fat mass (%)**			0.35
Baseline fat mass (% of BW)	−0.30	−0.32; −0.29	**<0.001**	
Age (years)	0.02	0.01; 0.03	**<0.001**	
Women	3.65	3.45; 3.86	**<0.001**	
Accumulated expense (1000€)	−0.25	−0.34; −0.16	**<0.001**	
Number of visits (n)	−0.17	−0.20; −0.14	**<0.001**	
**Model 4**	**Δ Muscle (%)**			0.35
Baseline muscle (% of BW)	−0.30	−0.31; −0.28	**<0.001**	
Age (years)	−0.06	−0.07; −0.06	**<0.001**	
Women	−2.42	−2.55; −2.30	**<0.001**	
Accumulated expense (1000€)	0.14	0.08; 0.19	**<0.001**	
Number of visits (n)	0.10	0.08; 0.12	**<0.001**	

β represents changes in outcomes for an increasing number of units of body weight, BMI and fat mass loss and muscle gain in the whole population. Bold numbers indicate statistical significance (*p* < 0.05). CI, Confidence Intervals; BMI, Body Mass Index; BW, Body Weight.

**Table 3:** Logistic regression models of the success of body weight, BMI and fat mass loss and muscle gain.

	OR	C.I.	*p*-Value	R^2^
**Model 1**	**10 kg of weight loss (kg)**			0.24
Baseline body weight (kg)	1.08	1.08; 1.09	**<0.001**	
Age (years)	1.00	0.99; 1.01	0.619	
Women	1.19	0.98; 1.43	0.076	
Accumulated expense (€)	1.00	1.00; 1.00	**<0.001**	
Number of visits (n)	1.13	1.10; 1.16	**<0.001**	
Restart (yes)	0.13	0.08; 0.21	**<0.001**	
**Model 2**	**3 kg/m^2^ of BMI loss (kg/m^2^)**			0.24
Baseline BMI (kg/m^2^)	1.35	1.32; 1.38	**<0.001**	
Age (years)	1.00	0.99; 1.00	0.267	
Women	0.78	0.67; 0.90	**0.001**	
Accumulated expense (€)	1.00	1.00; 1.00	**<0.001**	
Number of visits (n)	1.13	1.10; 1.16	**<0.001**	
Restart (yes)	0.14	0.10; 0.21	**<0.001**	
**Model 3**	**5% of fat mass loss (%)**			0.11
Baseline fat mass (% of BW)	1.09	1.08; 1.10	**<0.001**	
Age (years)	0.99	0.99; 1.00	**0.006**	
Women	0.25	0.21; 0.30	**<0.001**	
Accumulated expense (€)	1.00	1.00; 1.00	**0.004**	
Number of visits (n)	1.08	1.06; 1.11	**<0.001**	
Restart (yes)	0.27	0.22; 0.34	**<0.001**	
**Model 4**	**2% of muscle gain (%)**			0.18
Baseline muscle (% of BW)	0.80	0.79; 0.82	**<0.001**	
Age (years)	0.96	0.95; 0.97	**<0.001**	
Women	0.18	0.15; 0.21	**<0.001**	
Accumulated expense (€)	1.00	1.00; 1.00	**<0.001**	
Number of visits (n)	1.10	1.07; 1.12	**<0.001**	
Restart (yes)	0.20	0.15; 0.26	**<0.001**	

Bold numbers indicate statistical significance (*p* < 0.05). CI, Confidence Intervals; BMI, Body Mass Index; BW, Body Weight.

**Table 4:** Description of population subgroups or clusters.

	Cluster 1	Cluster 2	*p*-Value	Variable Contribution
**n**	**5528**	**1885**		
Age (years)	47.5 (10.5)	44.8 (10.0)	**<0.001**	1
Women (%)	5145 (93.1)	663 (35.2)	**<0.001**	0.78
Total days of follow-up (days)	109.1 (113.3)	111.2 (92.5)	0.484	0.94
Number of visits (n)	5.3 (4.6)	5.5 (4.2)	**0.026**	0.94
Doses per day (n)	4.8 (0.8)	5.0 (0.6)	**<0.001**	0.79
Accumulated expense (€)	2003.0 (1703.9)	2139.3 (1541.3)	**0.002**	0.86
Baseline weight (kg)	78.0 (8.1)	98.7 (11.4)	**<0.001**	0.79
Δ body weight (kg)	−5.0 (4.8)	−9.9 (7.3)	**<0.001**	0.70
Δ body weight (%)	−6.3 (5.8)	−10.0 (7.1)	**<0.001**	0.58
Baseline BMI (kg/m^2^)	29.4 (2.8)	32.5 (3.2)	**<0.001**	0.79
Δ BMI (kg/m^2^)	−1.9 (1.9)	−3.3 (2.5)	**<0.001**	0.70
Baseline fat mass (% of BW)	40.2 (5.7)	40.9 (7.1)	**<0.001**	0.67
Δ fat mass (% of BW)	−3.5 (3.6)	−6.2 (5.0)	**<0.001**	0.61
Δ fat mass (% of baseline value)	−8.2 (8.4)	−14.9 (11.5)	**<0.001**	0.53
Baseline visceral fat (% of BW)	6.1 (1.7)	10.8 (3.8)	**<0.001**	0.69
Δ visceral fat (% of BW)	−0.4 (0.8)	−1.6 (1.7)	**<0.001**	0.59
Δ visceral fat (% of baseline value)	−6.5 (8.9)	−12.7 (28)	**<0.001**	0.50
Baseline muscle mass (% of BW)	30.1 (3.9)	29.7 (4.6)	**0.001**	0.76
Δ muscle (% of BW)	2.0 (2.1)	3.7 (3.1)	**<0.001**	0.66
Δ muscle (% of baseline value)	6.9 (7.7)	13.3 (11.8)	**<0.001**	0.58

BMI, Body Mass Index; BW, Body Weight.

## Data Availability

The datasets presented in this article are not readily available because of data protection regulations. Data can be made available in a de-identified manner to researchers upon reasonable request (to the extent allowed by the registry’s data protection agreement). Requests to access these datasets should be directed to the investigator, Daniel de Luis (dluisro@saludcastillayleon.es).

## Supplementary Materials

The following supporting information can be downloaded at: https://www.mdpi.com/article/10.3390/jpm15060251/s1; Table S1: Comparison of acute changes produced in body weight by slimming diets with different macronutrient distributions; Table S2: Relative risk of anthropometric exits (hazard ratios and 95% CI) according to related-co variates in PNK cohort.
