# Supplementary material for: Phenotype-Driven Variability in Longitudinal Body Composition Changes After a Very Low-Calorie Ketogenic Intervention: A Machine Learning Cluster Approach
Source: J Pers Med. 2025 Jun 14;15(6):251. doi: 10.3390/jpm15060251 (PMC12193932; doi:10.3390/jpm15060251)
Supplement: Supplementary file 1 [file jpm-15-00251-s001.zip › Supplemental Table S1.pdf]

Supplemental Table S1. Comparison of acute changes produced in body weight by slimming diets with different macronutrient distributions

Diet characteristics	Participant characteristics	Duration	Δ Total weight loss kg/% (SD)	Δ Average weekly weight loss kg/%/week	Fat mass loss kg/% (SD)	Fat-free mass loss kg/% (SD)	(Ref.) year
**Manipulation of macronutrient content**							
**1. Energy deficit of 600 kcal/d with high or low-fat content**							
20-25% fat	389 ♀/♂	8 wk	6.9 (3.4) kg	0.69 kg/wk	5.4 (3.1) kg	1.5 (2.3) kg	(47) 2006
40-45% fat	382 ♀/♂		6.6 (3.5) kg	0.65 kg/wk	5.0 (4.1) kg	1.6 (3.7) kg	
**2. Energy deficit of 30% with high or low glycemic index**							
GI=60-65	16 ♀/♂	8 wk	5.3 (2.6) %	0.66 %/wk	13.1 (8.5) %	1.3 (3.9) %	(48) 2008
GI=40-45			7.5 (2.9) %	0.94 %/wk	14.8 (5.8) %	3.5 (3.3) %	
**3. Energy deficit of 500 kcal/d with high protein or high CHO intake**							
High protein	11 ♀	10 wk	9.2 (1.5) kg	0.92 kg/wk	7.9 (1.1)	0.2-0.6 kg (*NA*)	(49)2003
High CHO	11 ♀		4.8 (1.1) kg	0.48 kg/wk	4.2 (0.7)		
**4. VLCKDs with 600-1500 kcal/d, based on meal replacements and natural foods with different fat content and supplementation**							
40g EVO, phytoextracts	106 ♀/♂	6 wk	6.8 (*NA*) kg	1.13 kg/wk	6.2 (*NA*) %	*NA*	(50) 2011
30g EVO, n-3 PUFAs	135 ♀/♂	1 mo	6.6 (*NA*) kg	1.52 kg/wk	2.17 (*NA*) %	+2.2 (*NA*) %	(51)2022
10g EVO, n-3 PUFAs	20 ♀/♂	4 mo	20.8 (*NA*) kg	1.18 kg/wk	16.5 (*NA*) kg	3.8 (*NA*) kg	(80) 2017
**5. VLCD with 800-1000 kcal/d based on high-protein meal replacements and raw vegetables**							
	145 ♀/♂	8 wk	11.1 (3.8) kg	1.39 kg/wk	8.4 (4.2) kg	*NA*	(52) 2012
**Manipulation of the dietary pattern**							
**6. Energy deficit of 30% with high n-3 PUFA and antioxidant intake, 7 low GI meals/d**							
	27 ♀/♂, MetS	6 mo	9.3 (3.0) kg	0.34 kg/wk	7.9 (*NA*) kg	1.6 (*NA*) kg	(53) 2013
**7. Energy deficit of 30% with fatty fish, lean fish or fish oil**							
Cod 3d/wk	70, ♀/♂	8 wk	5.4 (2.7) kg	0.68 kg/wk	3.4 (2.6) kg	2.0 (2.4) kg	(54) 2007
Salmon 3d/wk	74, ♀/♂		5.5 (3.3) kg	0.69 kg/wk	3.5 (2.3) kg	2.0 (1.7) kg	
Fish oil capsules 6/d	68, ♀/♂		5.4 (3.2) kg	0.68 kg/wk	3.7 (2.3) kg	1.6 (1.7) kg	

**8. Energy deficit of 30% with legumes, animal protein or fatty fish**							
Legumes 4/wk	8 ♂		8.3 (2.9) %	1.04 %/wk	15.1 (6.6) %	5.0 (2.6) %	
Fatty fish 3d/wk	8 ♂	8 wk	6.4 (2.6) %	0.80 %/wk	14.3 (7.6) %	3.4 (2.5) %	(55) 2009
High animal protein	9 ♂		8.4 (1.2) %	1.05 %/wk	18.6 (3.3) %	4.9 (1.6) %	
**9. Mediterranean diet with 500 kcal/d energy deficit**							
	133, ♀/♂	3 mo	6.75 (*NA*) kg	0.52 kg/wk	-3.15 (%)	+2,9 (%)	(51) 2022
**Manipulation of meal timing**							
**10.Intermittent or continuous calorie restriction with an average of 20% energy deficit/wk**							
ICR, 75% CR 2 days/wk	49, ♀/♂	12 wk	7.1 (0.7) %	0.59 %/wk	*NA*	*NA*	(56) 2018
CCR, 20% CR 7 days/wk			5.2 (0.6) %	0.43 %/wk	*NA*	*NA*	
**11. Alternate day fasting with 75% energy deficit every second day, eating only lunch, dinner or 3 mini-meals**							
ADF, lunch only	20, ♀/♂		3.5 (0.4) kg	0.44 kg/wk	*NA*	*NA*	
ADF, dinner only	19, ♀/♂	8 wk	4.1 (0.5) kg	0.51 kg/wk	*NA*	*NA*	(57)2014
ADF, 3 mini-meals	20, ♀/♂		4.0 (0.5) kg	0.50 kg/wk	*NA*	*NA*	
**12. Time-restricted eating with an 8-hour eating window and *ad libitum* energy intake**							
	20, ♀/♂	12 wk	3.6 kg	0.3 kg/wk	1.7 kg	1.4 kg	(58) 2020
**13. Energy deficit of 10% eating CHOs and proteins mostly at lunch or mostly at dinner**							
Diurnal CHO/nocturnal protein	21	8 wk	3.29 (2.47) kg	0.41 kg/wk	2.6 (2.0) kg	0.7 (1.8) kg	(59) 2014
Nocturnal CHO/diurnal protein	19		3.81 (3.67) kg	0.47 kg/wk	2.9 (2.7) kg	0.9 (1.5) kg	

kcal/d: kilocalories/day; wk: week; mo: month; SD, Standard Deviation; GI, Glycemic Index; CHO, Carbohydrates; VLCKD, Very Low-Calorie Ketogenic Diet; EVO, Extra-Virgin Olive Oil; n-3 FA, Omega 3 Polyunsaturated Fatty Acids; NA, Not Available; VLCD, Very Low-Calorie Diet; MetS, Metabolic Syndrome; MD, Mediterranean Diet; ICR, Intermittent Calorie Restriction; CCR, Continuous Calorie Restriction; ADF, Alternate-Day Fasting
