# Supplementary material for: Phenotype-Driven Variability in Longitudinal Body Composition Changes After a Very Low-Calorie Ketogenic Intervention: A Machine Learning Cluster Approach
Source: J Pers Med. 2025 Jun 14;15(6):251. doi: 10.3390/jpm15060251 (PMC12193932; doi:10.3390/jpm15060251)
Supplement: Supplementary file 1 [file jpm-15-00251-s001.zip › Supplemental Table S2.pdf]

Supplemental Table S2. Relative risk of anthropometric exits (hazard ratios and 95% CI) according to related-co variates in PNK cohort.

	Total			Cluster1			Cluster2			
**Model**	**Cases/people-y**	**HR (95% CI)**	**p-value**	**Cases/people-y**	**HR (95% CI)**	**p-value**	**Cases/people-y**	**HR (95% CI)**	**p-value**	**p-interaction**
**10 kg of weight loss**	1518/1010.11			754/755.75			764/254.35			**<0.001**
Cluster 2 (1 as ref.)		2.258 (1.729-2.950)	**<0.001**							
Total days of follow-up		1.002 (1.000-1.003)	**0.009**		1.000 (0.998-1.002)	0.686		1.000 (0.998-1.002)	0.825	
Baseline weight (kg)		1.037 (1.032-1.043)	**<0.001**		1.073 (1.062-1.085)	**<0.001**		1.020 (1.012-1.028)	**<0.001**	
Women (men as ref.)		1.072 (0.903-1.273)	0.425		0.875 (0.645-1.188)	0.392		0.867 (0.692-1.085)	0.212	
Accumulated expense (€)		1.000 (1.000-1.000)	**<0.001**		1.000 (1.000-1.000)	**<0.001**		1.000 (1.000-1.000)	**<0.001**	
Time		1.201 (1.164-1.238)	**<0.001**		1.243 (1.187-1.302)	**<0.001**		1.179 (1.125-1.236)	**<0.001**	
Relapse (no as ref.)		0.057 (0.035-0.093)	**<0.001**		0.062 (0.032-0.120)	**<0.001**		0.066 (0.033-0.135)	**<0.001**	
**5% of fat mass loss**	4247/1010.11			2930/755.75			1317/254.35			**<0.001**
Cluster 2 (1 as ref.)		1.266 (1.090-1.470)	**0.002**							
Total days of follow-up		0.997 (0.996-0.998)	**<0.001**		0.996 (0.995-0.997)	**<0.001**		0.998 (0.996-1.000)	**0.034**	
Baseline fat mass (% of BW)		1.021 (1.015-1.027)	**<0.001**		1.028 (1.021-1.036)	**<0.001**		1.011 (1.001-1.022)	**0.038**	
Women (men as ref.)		0.730 (0.655-0.814)	**<0.001**		0.685 (0.577-0.813)	**<0.001**		0.761 (0.649-0.892)	**0.001**	
Accumulated expense (€)		1.000 (1.000-1.000)	**<0.001**		1.000 (1.000-1.000)	**<0.001**		1.000 (1.000-1.000)	**<0.001**	
Time		1.171 (1.147-1.196)	**<0.001**		1.206 (1.174-1.238)	**<0.001**		1.114 (1.071-1.159)	**<0.001**	
Relapse (no as ref.)		0.139 (0.117-0.166)	**<0.001**		0.153 (0.125-0.187)	**<0.001**		0.128 (0.091-0.181)	**<0.001**	
**3 kg/m² of BMI loss**	2041/1010.11			1212/755.75			829/254.35			**<0.001**
Cluster 2 (1 as ref.)		1.692 (1.354-2.116)	**<0.001**							
Total days of follow-up		1.000 (0.999-1.001)	0.862		0.999 (0.997-1.000)	0.074		1.000 (0.998-1.002)	0.900	
Baseline BMI (kg/m²)		1.164 (1.146-1.184)	**<0.001**		1.233 (1.204-1.262)	**<0.001**		1.091 (1.063-1.121)	**<0.001**	
Women (men as ref.)		0.973 (0.845-1.119)	0.700		0.847 (0.662-1.084)	0.187		0.856 (0.707-1.037)	0.112	
Accumulated expense (€)		1.000 (1.000-1.000)	**<0.001**		1.000 (1.000-1.000)	**<0.001**		1.000 (1.000-1.000)	**<0.001**	
Time		1.179 (1.147-1.212)	**<0.001**		1.206 (1.162-1.252)	**<0.001**		1.151 (1.099-1.204)	**<0.001**	
Relapse (no as ref.)		0.066 (0.045-0.098)	**<0.001**		0.074 (0.046-0.119)	**<0.001**		0.065 (0.033-0.128)	**<0.001**	
**2% of muscle gain**	3229/1010.11			2093/755.75			1136/254.35			**<0.001**
Cluster 2 (1 as ref.)		1.390 (1.169-1.653)	**<0.001**							
Total days of follow-up		0.999 (0.998-1.000)	**0.008**		0.997 (0.996-0.999)	**<0.001**		0.999 (0.997-1.001)	0.231	
Baseline muscle (% of BW)		0.932 (0.922-0.943)	**<0.001**		0.895 (0.881-0.910)	**<0.001**		0.973 (0.955-0.991)	**0.003**	
Women (men as ref.)		0.594 (0.526-0.670)	**<0.001**		0.468 (0.380-0.578)	**<0.001**		0.662 (0.561-0.782)	**<0.001**	
Accumulated expense (€)		1.000 (1.000-1.000)	**<0.001**		1.000 (1.000-1.000)	**<0.001**		1.000 (1.000-1.000)	**<0.001**	
Time		1.164 (1.138-1.191)	**<0.001**		1.195 (1.159-1.231)	**<0.001**		1.119 (1.075-1.166)	**<0.001**	
Relapse (no as ref.)		0.100 (0.079-0.127)	**<0.001**		0.111 (0.084-0.148)	**<0.001**		0.095 (0.061-0.146)	**<0.001**	

Bold numbers indicate statistical significance ($p < 0.05$). CI, Confidence Intervals; HR, Hazard Ratio; BW, Body Weight; BMI, Body Mass Index.
P-interaction between cluster#days of treatment.
